# Infusing Planetary Health into Clinical Teaching: A Curriculum-Wide Approach To Sustainability in Medical Education

**DOI:** 10.1007/s40670-026-02667-x

**Published:** 2026-03-03

**Authors:** Matti L Gild, Jacinta Wong, Naomi Staples, Margaret Schnitzler, Karen M Scott

**Affiliations:** 1 Department of Endocrinology and Diabetes, Royal North Shore Hospital, Sydney, Australia; 2 Sydney Medical School, University of Sydney, Sydney, Australia; 3 Royal North Shore Hospital, Sydney, Australia; 4 Education Office, Sydney Medical School, University of Sydney, Sydney, Australia; 5 Department of Colorectal Surgery, Royal North Shore Hospital, Sydney, Australia; 6 Specialty of Child and Adolescent Health, University of Sydney, Sydney, Australia

## Abstract

We describe a co-designed innovative planetary health curriculum using an infusion model embedded across a four-year graduate medical program. Content about sustainability in healthcare is linked to clinical learning, encouraging students to view environmental responsibility as integral to good medical practice.

Climate change is widely recognised as the greatest global health threat of the 21st century. The healthcare sector is both a frontline responder and a significant contributor to environmental degradation with the sector itself responsible for 7% of Australia’s carbon footprint [1]. Sustainability in healthcare is therefore not only about reducing environmental impact, but also about supporting high-quality, resilient health systems that can meet current needs without compromising health. Preparing future health professionals to practise sustainably requires more than add-on lectures—it requires systemic curriculum integration. Various strategies have been developed by health professional programs to include planetary health (PH) teaching into their curricula, such as the co-development of a PH organ system map [2]. In 2021 University of Sydney Medical Program staff and students co-designed an innovative planetary health education model using a curriculum-wide infusion approach.

Rather than delivering sustainability through a stand-alone module, this approach embeds relevant content into existing clinical teaching across the four-year graduate-entry program. Early in the program, students are introduced to the concept of planetary health as part of their professional responsibility, laying the foundation for critical engagement throughout their training. In the cardiology block, for example, students examine the link between extreme heat and increased cardiovascular admissions; during renal teaching, the environmental impacts of dialysis are discussed and short videos explain significant water usage during dialysis, with concrete examples, including visual images of two bathtubs of water which are required for each dialysis session. These analogies assist students to appreciate the magnitude of the problem. Sustainability content is reinforced through a visual tagging system on the program’s learning management system; for example, high-carbon clinical interventions are marked with a ‘carbon footprint’ icon to prompt reflection. Podcasts developed by senior students extend learning beyond the classroom and promote peer engagement.

Co-design with students was a key component of this curriculum innovation. To produce such resources, four Year 2 students with an interest in climate change and environmental health issues became ideal collaborators for integrating sustainability principles. Avoiding traditional ‘top-down’ approaches where faculty alone determine content, academics met regularly with the students, who as design partners assisted in identifying priority topics and creative solutions. Students articulated sustainability concepts by developing short animated videos covering themes such as colorectal cancer and dietary risks, and minimising arterial blood gas usage. These animations served as educational ‘artefacts’ to inform curriculum integration within the medical program’s existing thematic weeks.

The infusion model is fundamentally scalable, as it leverages existing curricular structures and requires minimal additional teaching time. While topic selection and examples were tailored to the local curriculum, the underlying principles—co-design, clinical contextualisation, and short, targeted learning resources—are transferable across medical schools and health professional programs more broadly. This approach aligns with emerging planetary health education frameworks that advocate for longitudinal, integrated learning rather than stand-alone modules. Other international models emphasise this same approach to student-led, bottom-up planetary health initiatives, demonstrating that co-design and alignment with existing competency frameworks can enable meaningful integration [3].

Initial evaluation was performed regarding the impact of the infused planetary health curriculum and its influence on students’ interest in sustainability and perception of their learning. In 2022 all Year 1 students were invited via email to complete an online end-of-year survey, hosted on the Qualtrics^™^ survey platform. The survey contained five four-point Likert-scale items (strongly agree-strongly disagree) about sustainability teaching, which were analysed using descriptive statistics. The survey, completed by 76% of students (202/267), revealed strong student interest in sustainability in healthcare and strong support for integrating it into the medical program. Most students agreed (54%) or strongly agreed (39%) that environmental sustainability in healthcare is important. Similarly, 85% supported incorporating sustainability teaching into the curricula and 74% believed there should be more teaching about sustainability. Following implementation of the infusion-based curriculum, 83% felt their understanding of sustainability in healthcare had increased and 70% reported a greater interest in sustainability in healthcare.

External evaluation through the Planetary Health Report Card (PHRC) was performed in 2025. This student-led international evaluation framework assesses how health professional schools address planetary health within their curricula, research, community engagement, and institutional sustainability practices. Our curriculum was evaluated favourably and was awarded an A- [4].

The survey results highlight that students in our medical program view sustainability as a critical issue for healthcare and strongly support its inclusion in their training. The infusion approach allows sustainability to be normalised and contextualised within each discipline, avoiding content overload while reinforcing its broad clinical relevance. Rather than treating planetary health as an isolated topic, students encounter it repeatedly, applied in real-world medical decision-making. Longitudinal research could explore the impact on changes in students’ beliefs and clinical practice. Nevertheless, the successful PHRC result benchmarks the infusion approach. Table 1 provides tips for infusing planetary health in health professional programs.

**Table 1:** 5 tips for infusing planetary health content in health-related programs

Tips	Examples
Embed planetary health into existing clinical blocks rather than a stand-alone module	Carbon cost of full blood count when concurrently learning haematology
Introduce planetary health early and reinforce longitudinally as a vertical theme	Heat and myocardial infarction risk in cardiology block in Year 1, then repeat when learning about cardiac failure in Year 2
Use clinically relevant, concrete examples	Water use in renal dialysis is equivalent to 2 full bathtubs of water
Enhance visibility of planetary health content with icons	
Select common topics	Carbon footprint of asthma puffers, differences in carbon emissions with anaesthetic gases

This infusion strategy, first pioneered by Mount Sinai Medical School and subsequently adopted by other institutions [5], is an effective educational strategy that promotes a longitudinal thread that connects environmental awareness with systems thinking and ethical healthcare practice. Infusion requires academic coordination but minimal additional teaching time. In addition, by positioning students as active design partners rather than passive recipients, co-design ensures educational resources are directly responsive to learner needs, preferences, and contemporary perspectives [6]. Utilising existing case-based and systems-based structures, planetary health becomes a lens through which clinical content is interpreted rather than a separate subject to be learned.

Health professional educators are uniquely positioned to influence how the next generation of clinicians understand their role in a rapidly changing world. Infusing planetary health throughout the curriculum signals that sustainability is not an optional extra, but a core component of medical competence. The value of short, targeted content delivered through innovative methods may help maintain interest and ensure that planetary health remains a visible and integral part of health professional education. The infusion-based approach is inherently scalable, as it leverages existing curricular structures and can be readily adapted across institutions, countries and health disciplines. This curriculum innovation represents a comprehensive approach to preparing future health professionals to address the challenges of planetary health, equipping students with the knowledge and skills necessary to practise environmentally sustainable health.
